# Automated facial coding software outperforms people in recognizing neutral faces as neutral from standardized datasets

**DOI:** 10.3389/fpsyg.2015.01386

**Published:** 2015-09-11

**Authors:** Peter Lewinski

**Affiliations:** The Amsterdam School of Communication Research, Department of Communication Science, University of AmsterdamAmsterdam, Netherlands

**Keywords:** non-verbal communication, facial expression, face recognition, neutral face, automated facial coding

## Abstract

Little is known about people’s accuracy of recognizing neutral faces as neutral. In this paper, I demonstrate the importance of knowing how well people recognize neutral faces. I contrasted human recognition scores of 100 typical, neutral front-up facial images with scores of an arguably objective judge – automated facial coding (AFC) software. I hypothesized that the software would outperform humans in recognizing neutral faces because of the inherently objective nature of computer algorithms. Results confirmed this hypothesis. I provided the first-ever evidence that computer software (90%) was more accurate in recognizing neutral faces than people were (59%). I posited two theoretical mechanisms, i.e., smile-as-a-baseline and false recognition of emotion, as possible explanations for my findings.

## Introduction

Recognizing a neutral face as neutral is vital in social interactions. By virtue of “expressing” “nothing” (for a separate discussion on faces “expressing” something, see Russell and Fernández-Dols, 1997), a neutral face should indicate lack of emotion, e.g., lack of anger, fear, or disgust. This article’s inspiration was the interesting observation that in the literature on facial recognition, little attention has been paid to neutral face recognition scores of human raters. Russell (1994) and Nelson and Russell (2013), who provided the two most important overviews on the topic, did not include or discuss recognition rates of lack of emotion (neutral) in neutral faces. They provided overviews of matching scores (i.e., accuracy) for six basic emotions, but they were silent on the issue of recognition accuracy of neutral faces.

A distinct lack of articles that explicitly report accuracy scores for recognition of neutral face could explain the silence of researchers in this field. One notable exception is the *Amsterdam Dynamic Facial Expression Set* (ADFES; van der Schalk et al., 2011), where the authors provide an average matching score of 0.67 for their neutral faces. This score is considerably low when one considers that an average for six basic emotions is also in this range ( 0.67, see Nelson and Russell, 2013, Table A1 for datasets between pre-1994 and 2010).

In this paper, I demonstrate a fascinating effect on the recognition of non-expressive, neutral faces both by humans and by software, though I can only speculate as to its theoretical mechanisms. I provide the first evidence that computer software is better in recognizing neutral faces than people are. I open up a potentially productive new area for studying the precise mechanism behind my findings, and I entertain speculation on two possible causes for my findings, i.e., smile-as-a-baseline and false recognition of emotion. In addition, I note in my discussion that independently of the exact mechanism, this finding already has practical implications.

In the current paper, I attempt to fill a gap in the literature regarding the analysis of recognition accuracy of neutral faces from secondary data of human raters and an “objective rater.” I define this objective rater as automated facial coding (AFC) software. Therefore, I compare the human versus software accuracy in recognizing neutral faces (i.e., lack of emotion) in clearly neutral images of a face. The use of such objective rater could become a standard in the field of non-verbal communication from facial expressions.

### Objective Rater

I assume throughout that the computer software is an objective rater because it follows the same coding schema (i.e., an algorithm) for every rating. Technically, software of this type cannot deviate from the algorithm and cannot take into account extraneous information, e.g., a social context or situation. Furthermore, software does not have personal biases stemming from age, culture, or gender. In short, computer software cannot display individual differences in recognizing emotions. To illustrate, I submit a far-fetched example. Studies on recognition of emotionally neutral faces in clinically depressed patients have revealed (e.g., Leppänen et al., 2004) that these individuals perform worse (are less accurate and slower) in recognizing neutral faces than healthy participants. Computer software cannot be depressed or otherwise experience emotional or cognitive abnormalities as humans can. Thus, most importantly, I argue that the software has no specific incentive to over-detect somehow ambiguous situations (such as neutral faces).

Furthermore, as explained below, the AFC software and human raters had essentially to perform the same task, i.e., to choose one target label (neutral) out of a single, unvarying choice set. A training set of ~10,000 images is extremely small, if it is compared to an average number of faces/expressions seen (both consciously and unconsciously) by an average person aged 30 years. But in the context of comparing human to software, the human rater arguably still has a much richer training data set (speaking figuratively) than the particular AFC software tested in this paper. It was not possible to locate a reference discussing how many faces/expressions an average, healthy adult sees by age 30, but this perhaps goes into trillions of instances, and thus AFC software should be no match to human recognition, but it nevertheless might be. This is likely because a human system is not “software” that just needs more instances of the same stimuli to recognize it correctly; instead, a human system likely has many recognition biases. Based on the above-mentioned reasoning, my hypothesis states that human raters will have significantly lower accuracy recognition rates than AFC software.

## Materials and Methods

To test my hypothesis, I gathered a representative sample of neutral faces from standardized datasets. I then computed human accuracy scores for these faces. Next, I analyzed those neutral faces with AFC software – FaceReader (Noldus, 2014) – and computed FaceReader’s accuracy scores. Finally, I compared the human and FaceReader performance in recognizing neutral faces. I report how I determined sample size and all study measures in the sections that follow.

### Neutral Faces

**FIGURE 1:**
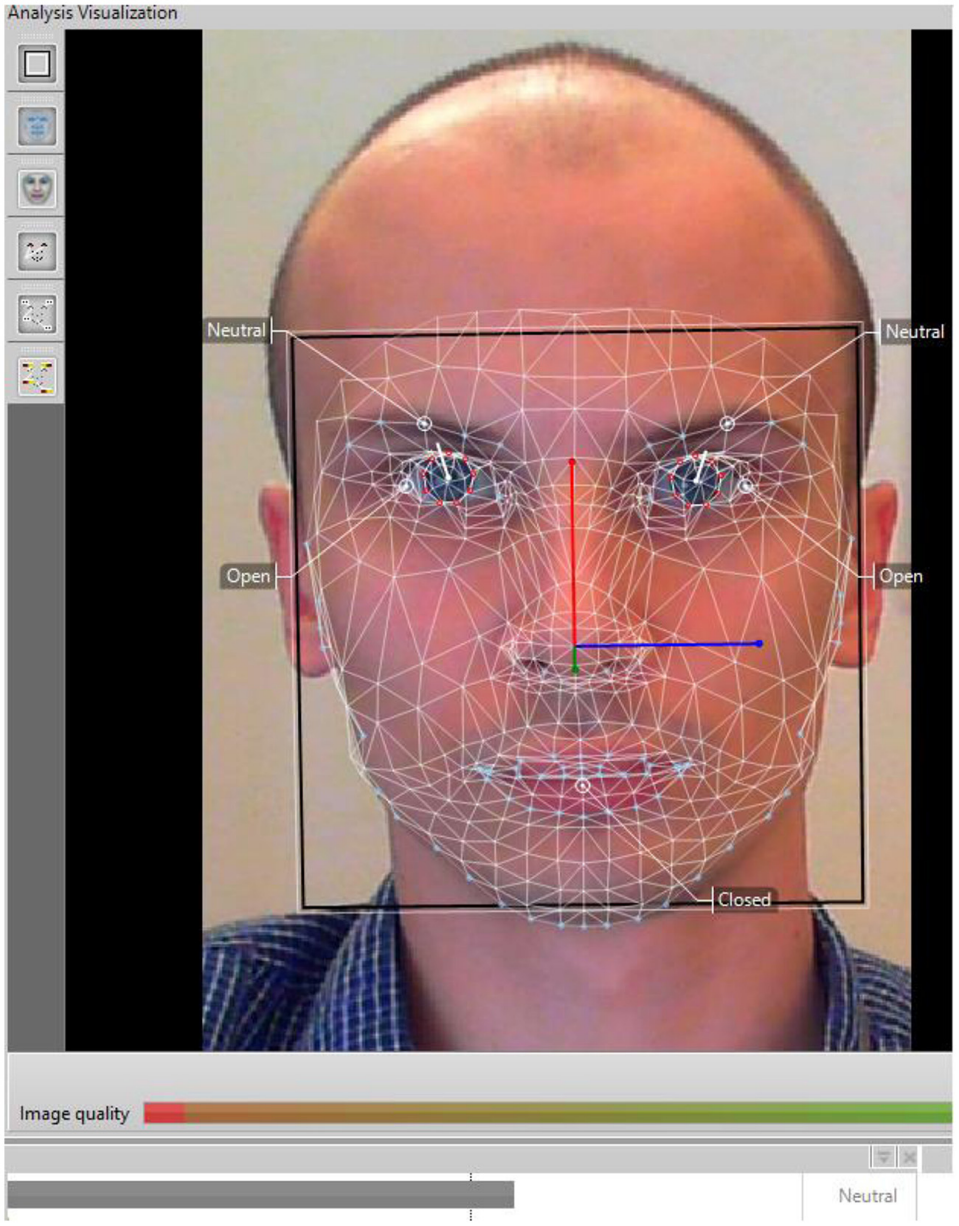
**FaceReader analysis of a neutral face**.

I used all available images of neutral faces in both *Karolinska Directed Emotional Faces* (KDEF; Goeleven et al., 2008) and *Warsaw Set of Emotional Facial Expression Pictures* (WSEFEP; Olszanowski et al., 2015) datasets. KDEF is a typical dataset with emotional faces, including baseline, that is, neutral images. See **Figure 1** for a typical neutral face image. WSEFEP is a dataset that closely replicates the KDEF methodology of gathering faces, i.e., it contains close-up, front-facing, light-adjusted images of people’s faces. The KDEF dataset is a standard dataset in facial expression and AFC research and a popular choice with researchers, being cited over 160 times. Importantly, this choice was also made because KDEF was included in the original training set of the AFC software and WSEFEP was not. This distinction allowed for testing whether this factor could explain potential differences.

#### Actors Posing a Face

In addition, during the creation of both datasets, the actors expressing the emotion (or posing a neutral face) received specific procedural instructions and underwent extensive training. Thus, consistency and standardization justified our selections of KDEF and its replication, WSEFEP. There were 70 neutral faces in KDEF (50% women) and 30 in WSEFEP (53% women), for a total of 100 images. The actors from those 100 images were specifically instructed to pose a neutral face (see Lundqvist et al., 1998 for definition of a neutral face) by the creators of the respective datasets.

Nevertheless, I sought to assure myself that the faces were indeed neutral. Therefore, the images were coded by a certified Facial Action Coding System (FACS) coder (Ekman et al., 2002) to identify if there could be any facial movement [so-called Action Unit, (AU)] indicative at least partially of an emotion. None of the images contained significant AU (or combinations of AUs), which I defined as part of a basic emotion expression based on EMFACS-7 classification (Friesen and Ekman, 1983). I note that it is unorthodox to use FACS (Ekman et al., 2002, p. 10) to “code” neutral still images, however, the Investigator’s Guide – part of the FACS manual – is not clear about this issue and in principle permits such coding. Further, Griffin (2014), among others, used a similar procedure in his studies to determine if a neutral face has a truly neutral expression. By using EMFACS-7 classification, the FACS manual’s Investigator’s Guide, and following up on Griffin (2014), I believe I have adopted the best approach to ensure truly neutral expressions.

#### Human Ratings of the Datasets

I manually downloaded the datasets and extracted from them the matching scores for the neutral faces (see Table A1 in the Appendix A). In both datasets, the matching scores were defined as “the percentage of observers who selected the predicted label” (Nelson and Russell, 2013, p. 9). I took these matching scores as proxy for accuracy of human recognition rates.

#### Human Face Categorization

The authors of both datasets asked the human judges to choose one label out of a list of six basic emotions (happy, surprised, angry, sad, disgusted, and fearful), a “neutral” or other option (KDEF – “indistinct”; WSEFEP – “acceptance,” “anticipation,” “other emotion”) when they saw a target face (*N*_KDEF_ = 490; *N*_WSEFEP_ = 210). In both datasets, the target faces showed a basic emotion expression or a neutral face. The order of presentation of all the faces was randomized in both datasets; furthermore, the human judges saw only a sub-sample of all possible target face images (to minimalize order effect as well as anchoring effect). See Appendix B for excerpts from the description of the two datasets on the judgment task for human coders.

### Automated Facial Coding Software – FaceReader

As an instance of AFC software, I used FaceReader (Noldus, 2014), a software tool that automatically and programmatically analyzes facial expression of emotion. An average recognition score of 89% over the six basic emotions was reported for FaceReader in den Uyl and van Kuilenberg (2005), revalidated to 88% in Lewinski et al. (2014a). This software has been available for scientific research since den Uyl and van Kuilenberg (2005). Researchers have used FaceReader in a multitude of contexts such as, but not limited to: human–computer interaction (Goldberg, 2014); social psychology (Chentsova-Dutton and Tsai, 2010); consumer science (Chan et al., 2014); advertising (Lewinski et al., 2014b); and multimedia research (Romero-Hall et al., 2014). Of more relevance to the current paper is the software’s specific use in assessing the role of recognition of emotional facial expressions in human raters only (Choliz and Fernandez-Abascal, 2012).

#### FaceReader Face Categorization

FaceReader works in three steps. First, it detects a face in the image. Next, it identifies 500 key landmark points in the face through Active Appearance Model (Cootes and Taylor, 2004), visualized as a 3D superimposed virtual mesh. In the last stage, it classifies the image according to how likely the emotion is present (or not) in a person’s face. A 3-layer, artificial neural network trained on more than 10,000 of instances of six basic emotions and neutral faces makes this classification possible. Then, the software can assign a label to each target face. FaceReader can choose from six basic emotions, a neutral label, as well as a “failed to recognize” option. Therefore, the software followed a very similar procedure to what human judges did. It had to choose a label for a target face out of six basic emotions, a neutral label or indicate it could not classify the face (failure). The number of classification choices is thus similar to the task that the human judges had, however, it could be argued that this is not a one-to-one task equivalency. See van Kuilenburg et al. (2005) for a detailed algorithmic description of this software. In addition, **Figure 1** provides a visualization of FaceReader analysis.

FaceReader’s emotion detection algorithm ranges from 0 to 1 for each basic emotion, plus neutral. Higher values indicate a greater likelihood that the person in the image or video experiences the target emotion (or lack thereof). I took this measure as a proxy for classification accuracy. It is technically impossible to compute matching scores for FaceReader as one might do with human raters because the number of “raters” is always *n* = 1, i.e., the software itself.

## Results

Human participants judged 100 images of neutral faces, while FaceReader analyzed the same 100 images. FaceReader successfully analyzed all the images (no “fail to detect”). An independent samples *t*-test was run to determine if there were differences in accuracy scores between humans and FaceReader. There was no homogeneity of variances, as assessed by Levene’s test for equality of variances (*p* < 0.0005). The accuracy scores were lower for humans (*M* = 0.59, *SD* = 0.23) than for FaceReader (*M* = 0.90, *SD* = 0.14), a statistically significant difference (*M* = -0.31, 95% CI [-0.37, -0.26]), [*t*(167.96) = 11.62, *p* < 0.0005]. Additionally, Cohen’s effect size value (*d* = 1.68) suggested a high practical significance (Cohen, 1988). People, on average, recognized 59 images as neutral out of a set of 100 neutral images; FaceReader recognized 90 images as neutral out of the same set. FaceReader outperformed humans by 31%, i.e., it accurately recognized 31 more images than humans did. See Table A1 in Appendix A in Supplementary Material for overview of accuracy scores for each image.

### Training Set

Potentially, inclusion or exclusion of KDEF and WSEFEP datasets in the software’s training set could bias the results, because software could possibly be better in recognizing a neutral face as neutral if it had previously seen it. According to the software developer, the KDEF dataset was included to train the software while the WSEFEP data set was not included. Furthermore, a number of unnamed datasets was also included in the training set, resulting in more than 10,000 images in the entire training set. Therefore, it is possible that the inclusion/exclusion in the training dataset could be a potential explanatory factor of the results reported above.

To demonstrate that this factor (inclusion vs. exclusion) does not bias the results, the same statistical tests as above were run separately on the KDEF and WSEFEP. Two separate independent samples *t*-tests were conducted, first only on the KDEF dataset (included in the training set) and then only on the WSEFEP (not included in the training dataset). As expected, there was a significant difference between accuracy scores of human coders and FaceReader in KDEF dataset only [*t*(138) = 11.12, *p* < 0.0005] as well as in WSEFEP dataset only [*t*(58) = 4.50, *p* < 0.0005], replicating the main results when the datasets are combined. Therefore, in this study, it does not matter for recognition of neutral faces if the datasets are included or not in the original training set for this particular AFC software.

## Discussion

I demonstrated that AFC software massively outperforms human raters in recognizing neutral faces, a finding with important, far-reaching implications. First, I recommend that recognition rates for neutral faces be reported in all future emotion recognition studies. Second, further study of why humans only recognize on average about 60% of neutral faces as neutral is crucial. This study did not test an explanatory mechanism. However, I offer some speculation regarding two theoretical reasons for humans’ surprisingly low performance in the sections below.

### Theoretical Implications

One explanation of my findings is the phenomenon of the smile-as-a-baseline. In contemporary society, the baseline, i.e., neutral, emotional expression might be a smile rather than a technically neutral face. Some researchers (see Lee et al., 2008) have presented evidence that neutral faces look threatening, or at least “negative.” This finding could shed light on why humans have so much trouble recognizing “nothing” in truly neutral faces. That is, people are socialized into seeing happiness (or at least some kind of emotion) in the course of interpreting other people’s emotions, acting upon that interpretation, and consequently relating better to other people.

Another explanation for my findings could be the phenomenon known as false recognition of emotion (see Fernández-Dols et al., 2008), which is, bizarrely, contradictory to the smile-as-a-baseline explanation. Fernández-Dols et al. (2008) found that semantic rather than perceptual context of the facial stimuli provokes erroneously perceiving a particular emotion in the facial expression. I add to this theory by showing relatively low accuracy for human raters (59%) and high accuracy for AFC software (90%) in recognizing neutral faces. Undoubtedly, AFC software has no semantic framework from which to draw; perhaps the lack of such a framework makes the software less biased in neutral face recognition (i.e., avoiding false-positive errors).

Today’s AFC software cannot interpret the surrounding semantic context of the face, whereas people perform that interpretation almost instinctively (Fernández-Dols et al., 2008). From the software’s “perspective,” seeing a face can only be a neutral experience, while a human might be scanning for an extra (contextual or semantic) layer of meaning in faces. However, it must be noted that the assumption behind this argument is that the difference between a perfect score of 100% and actual score of 59% (i.e., 41%) is accounted for by labeling the neutral face with another emotion label (e.g., anger, sadness, disgust, etc) or even non-emotional label. For example, a label of “indistinct” expression was provided in original labels in the KDEF dataset or “acceptance, anticipation” for images in the WSEFEP dataset. Thus, a new theoretical question arises if there would be a difference in the neutral score recognition if only emotional or only non-emotional labels were included in the original datasets. This can be investigated in future studies.

Also worth pointing out is that both AFC software and human raters had only a limited number of categories to choose from – both datasets used the so-called forced-choice method (see Russell, 1994 for criticism). Despite these conditions, the human recognition scores for neutral faces still fell short of Haidt’s criterion of 0.70–0.90 accuracy score, which is the threshold at which a particular emotion (or lack thereof) in the face could be considered universally recognizable (see Haidt and Keltner, 1999). Beyond these theoretical discussions, my findings also have some practical, real-world implications.

### Practical Implications

First, in the practice of professionals who judge others’ non-verbal behavior (e.g., police officers, judges, psychotherapists, etc), it must be highlighted that human observers are not usually sensitive enough to see a neutral face as neutral. This shortcoming may result in professionals acting based on incorrect assumptions (e.g., police officer subduing a pedestrian because of wrongly assuming that a face was not neutral but angry).

Second, with the advent of wearable devices such as Google Glass, the clear advantage of software in recognizing neutral faces might be exploited. Even though Google Glass has been discontinued as of the beginning of 2015, the genie is out of the bottle – similar powerful devices are expected to be available in the near future. Thus, considering the situation described in the previous paragraph, a police officer equipped with Google Glass could be more effective in executing their duties (in Dubai, this is already the case, as seen in Gulf News from May 20, 2014). Wearable tech like Google Glass that included a utility like AFC software could indicate when others have a neutral face, reducing the chances of police officers engaging in needless interventions, possibly reducing violence overall.

### Limitations

#### Image Quality

One possible limitation of my study is the use of AFC software as an “objective” rater. AFC software has been known, in principle, to code expressions slightly differently depending, for instance, on the positioning of the face in the picture, uneven saturation, or varying hue (see e.g., FaceReader manual; Noldus, 2014). As much as this is a valid argument, it is equally valid for human raters, as people would be similarly influenced by image quality in judging facial expression.

#### Face Morphology

Another possible limitation to our study is the morphology of the face itself (e.g., wrinkles, bulges, folds; see e.g., Hess et al., 2004). For example, some people exhibit a shape to the mouth that naturally – i.e., when not otherwise emoting – looks like a smile (curved up) or a frown (curved down). Hairy eyebrows, meanwhile, may also give the appearance of a frown. Because of differences in facial morphology, neutral affiliate faces are less readily confused with angry faces than are dominant faces (Hess et al., 2007). I did not control for such possible morphological differences as part of the study, any more than FACS did so in coding the images or in my selection of the images.

However, I argue that I did indeed control for these possible confounds by presenting the exactly same set of neutral faces to the AFC software as was presented to human raters. Any possible differences in image quality, related photo characteristics, or facial morphology were kept constant and were the same for both software and human raters. If the software were possibly “confused” by the quality of the photo or the morphology of the face, this factor would apply equally to the human raters. In any case, I deem this particular limitation unlikely due to the highly standardized nature of the image sets used (see Materials and Methods).

#### Posed vs. Spontaneous Expressions

On the theoretical level, the current manuscript investigates and focuses only on difference in perception (both by software and human coders) coming from datasets that had clear, posed, and prototypical expressions. Such sets are standard in the field because they allow for heightened control over the independent variable to which the participants are exposed (the stimuli itself), as well as helping to define what is meant by a particular emotion. Furthermore, this paper focuses only on neutral expressions, and in principle, the same issues of similarity between posed and spontaneous facial expressions of emotion likely do not apply to a study of neutral faces. It was perhaps never tested, but it is difficult to think of a theoretical or practical reason why there should be a difference in neutral face recognition based on whether it comes from a spontaneous or posed facial expressions dataset (e.g., there is no muscle movement in neutral faces as there is in the case of emotional expressions). The current paper investigates only neutral expression and thus the debate on posed vs. spontaneous expressions is likely not applicable to neutral expressions to the same degree it is to emotional expressions. However, future studies may indeed find it worthwhile to test software vs. human accuracy on spontaneous expressions datasets to test this assumption empirically.

#### Coding Task

My hope is that the clarifications in the introduction and methods sections on the procedure and internal working of the software, as well as the thorough description of the participants’ task, provided sufficient evidence that the task of software and human raters was similar in nature and that humans should inherently have an advantage over the software. However, I recognize that human judges in both the datasets and FaceReader software had slightly different recognition tasks, as the choice set varied across all three instances, and this might have biased results.

Nonetheless, it must be recognized that none of the existing datasets are constructed in exactly the same way. Human judges in KDEF and WSEFEP, as well as in other famous datasets (e.g., JACFEE, MSFDE, ADFES, RaFD, UCDSEE, FACES), varied in terms of at least (a) including human rating; (b) recognition procedure; and (c) inclusion of a “neutral” and/or “other” label. This is why it was not possible to include these other datasets, as only two datasets were identified that contained human scores for neutral faces and followed a protocol similar to what the AFC software follows. WSEFEP and KDEF met those criteria, hence their use in this paper. Furthermore, to evaluate other famous datasets, it would be necessary to access raw images from those datasets and have them judged by human coders, e.g., on a crowdsourcing platform. This task was deemed beyond the focus of the current paper.

#### Anchoring Effect

Another possible limitation of this study lies in using the “matching scores” (i.e., the accuracy) for human raters from the dataset itself (WSEFEP and KDEF). In both of the datasets, the raters were also judging other basic emotional expressions (to validate those datasets), as in repeated-measure experiments. See Olszanowski et al. (2015; WSEFEP) and Goeleven et al. (2008; KDEF) for more details. Even though each rater saw only a limited number of images to classify and the order was randomized, the possibility exists that rating other-than-neutral faces could have resulted in a so-called anchoring effect (e.g., see Russell, 1994). In other words, previously witnessed emotion could have influenced the recognition of the subsequent expression. Nevertheless, both KDEF and WSEFEP, which followed the KDEF methodology, are typical instances of facial expression datasets used widely in research. For the developers of those sets, it would be impractical to expose human raters to only one subset of images, as this would result in a gargantuan sample needed to judge those facial images. A possible solution to this issue would include presenting the subset of neutral images in random order to a number of independent judges recruited from crowdsourcing platforms (e.g., MTurk). I may well adopt that methodology in future studies.

## Conflict of Interest Statement

Peter Lewinski has worked – as a Marie Curie Research Fellow – for Vicarious Perception Technologies B.V., Amsterdam – an artificial intelligence company that develops FaceReader software for Noldus Information Technologies B.V. He is also a research fellow in ASCoR.
