# Supplementary material for: Automated facial coding software outperforms people in recognizing neutral faces as neutral from standardized datasets
Source: Front Psychol. 2015 Sep 11;6:1386. doi: 10.3389/fpsyg.2015.01386 (PMC4565996; doi:10.3389/fpsyg.2015.01386)
Supplement: Supplementary file 1 [file Data_Sheet_1.DOCX]

**Appendix A**

Table A1. Human vs FaceReader Accuracy

| Image ID | Sex | Face | Human Accuracy | N | FR Accuracy | Dataset |
| --- | --- | --- | --- | --- | --- | --- |
| AD_7885 | F | Neutral | 0.53 | 17 | 0.97 | WSEFEP |
| AG_0011 | M | Neutral | 0.65 | 17 | 0.14 | WSEFEP |
| DC_0014 | M | Neutral | 0.71 | 24 | 0.75 | WSEFEP |
| HW_0006 | M | Neutral | 0.40 | 20 | 0.28 | WSEFEP |
| JG_1226 | M | Neutral | 0.80 | 15 | 0.96 | WSEFEP |
| JS_0008 | F | Neutral | 0.84 | 19 | 0.94 | WSEFEP |
| KA_0003 | M | Neutral | 0.57 | 21 | 0.99 | WSEFEP |
| KL_0024 | F | Neutral | 0.65 | 26 | 0.60 | WSEFEP |
| KM_0017 | M | Neutral | 0.59 | 17 | 0.93 | WSEFEP |
| KO_0031 | F | Neutral | 0.78 | 27 | 0.89 | WSEFEP |
| KP_0082 | F | Neutral | 0.94 | 17 | 1.00 | WSEFEP |
| KS_2161 | F | Neutral | 0.63 | 27 | 0.96 | WSEFEP |
| MB_0026 | F | Neutral | 0.68 | 25 | 0.84 | WSEFEP |
| MG_0345 | M | Neutral | 0.76 | 17 | 0.99 | WSEFEP |
| MJ_0346 | F | Neutral | 0.48 | 23 | 0.58 | WSEFEP |
| MK_0001 | M | Neutral | 0.58 | 24 | 0.99 | WSEFEP |
| MK1_0007 | F | Neutral | 0.63 | 27 | 0.98 | WSEFEP |
| MR_0013 | F | Neutral | 0.67 | 21 | 0.94 | WSEFEP |
| MR1_0006 | F | Neutral | 0.65 | 23 | 0.99 | WSEFEP |
| MR2_0014 | M | Neutral | 0.71 | 14 | 0.94 | WSEFEP |
| MS_0004 | F | Neutral | 0.65 | 31 | 0.97 | WSEFEP |
| OG_6108 | F | Neutral | 0.85 | 20 | 0.99 | WSEFEP |
| PA_0006 | M | Neutral | 0.80 | 25 | 0.99 | WSEFEP |
| PB_0001 | M | Neutral | 0.50 | 26 | 0.99 | WSEFEP |
| PO_0015 | M | Neutral | 0.59 | 17 | 1.00 | WSEFEP |
| PS_0216 | F | Neutral | 0.52 | 21 | 0.80 | WSEFEP |
| RA_0057 | M | Neutral | 0.00 |  | 0.96 | WSEFEP |
| RB_0006 | M | Neutral | 0.50 | 20 | 0.99 | WSEFEP |
| SO_2188 | F | Neutral | 0.91 | 33 | 0.93 | WSEFEP |
| SS_0151 | F | Neutral | 0.81 | 21 | 0.93 | WSEFEP |
| F01 | F | Neutral | 0.84 | 447 | 1.00 | KDEF |
| F02 | F | Neutral | 0.54 | 447 | 0.93 | KDEF |
| F03 | F | Neutral | 0.74 | 447 | 1.00 | KDEF |
| F04 | F | Neutral | 0.58 | 447 | 0.94 | KDEF |
| F05 | F | Neutral | 0.82 | 447 | 0.92 | KDEF |
| F06 | F | Neutral | 0.92 | 447 | 1.00 | KDEF |
| F07 | F | Neutral | 0.86 | 447 | 0.90 | KDEF |
| F08 | F | Neutral | 0.61 | 447 | 0.86 | KDEF |
| F09 | F | Neutral | 0.55 | 447 | 0.94 | KDEF |
| F10 | F | Neutral | 0.41 | 447 | 0.43 | KDEF |
| F11 | F | Neutral | 0.29 | 447 | 0.61 | KDEF |
| F12 | F | Neutral | 0.27 | 447 | 0.93 | KDEF |
| F13 | F | Neutral | 0.91 | 447 | 0.97 | KDEF |
| F14 | F | Neutral | 0.27 | 447 | 0.93 | KDEF |
| F15 | F | Neutral | 0.18 | 447 | 0.63 | KDEF |
| F16 | F | Neutral | 0.77 | 447 | 0.97 | KDEF |
| F17 | F | Neutral | 0.64 | 447 | 1.00 | KDEF |
| F18 | F | Neutral | 0.63 | 447 | 0.96 | KDEF |
| F19 | F | Neutral | 0.89 | 447 | 0.97 | KDEF |
| F20 | F | Neutral | 0.21 | 447 | 0.73 | KDEF |
| F21 | F | Neutral | 0.27 | 447 | 0.64 | KDEF |
| F22 | F | Neutral | 0.47 | 447 | 0.95 | KDEF |
| F23 | F | Neutral | 0.66 | 447 | 0.90 | KDEF |
| F24 | F | Neutral | 0.77 | 447 | 0.92 | KDEF |
| F25 | F | Neutral | 0.24 | 447 | 0.99 | KDEF |
| F26 | F | Neutral | 0.77 | 447 | 0.99 | KDEF |
| F27 | F | Neutral | 0.45 | 447 | 0.93 | KDEF |
| F28 | F | Neutral | 0.61 | 447 | 0.89 | KDEF |
| F29 | F | Neutral | 0.82 | 447 | 0.97 | KDEF |
| F30 | F | Neutral | 0.00 | 447 | 0.93 | KDEF |
| F31 | F | Neutral | 0.60 | 447 | 0.88 | KDEF |
| F32 | F | Neutral | 0.53 | 447 | 0.91 | KDEF |
| F33 | F | Neutral | 0.42 | 447 | 0.87 | KDEF |
| F34 | F | Neutral | 0.68 | 447 | 0.98 | KDEF |
| F35 | F | Neutral | 0.58 | 447 | 0.83 | KDEF |
| M1 | M | Neutral | 0.75 | 447 | 0.97 | KDEF |
| M2 | M | Neutral | 0.53 | 447 | 0.95 | KDEF |
| M3 | M | Neutral | 0.38 | 447 | 0.92 | KDEF |
| M4 | M | Neutral | 0.64 | 447 | 0.94 | KDEF |
| M5 | M | Neutral | 0.64 | 447 | 0.90 | KDEF |
| M6 | M | Neutral | 0.83 | 447 | 1.00 | KDEF |
| M7 | M | Neutral | 0.72 | 447 | 0.92 | KDEF |
| M8 | M | Neutral | 0.81 | 447 | 0.96 | KDEF |
| M9 | M | Neutral | 0.61 | 447 | 0.99 | KDEF |
| M10 | M | Neutral | 0.86 | 447 | 0.96 | KDEF |
| M11 | M | Neutral | 0.95 | 447 | 0.74 | KDEF |
| M12 | M | Neutral | 0.65 | 447 | 0.94 | KDEF |
| M13 | M | Neutral | 0.83 | 447 | 0.99 | KDEF |
| M14 | M | Neutral | 0.73 | 447 | 0.87 | KDEF |
| M15 | M | Neutral | 0.41 | 447 | 0.87 | KDEF |
| M16 | M | Neutral | 0.64 | 447 | 0.99 | KDEF |
| M17 | M | Neutral | 0.64 | 447 | 0.84 | KDEF |
| M18 | M | Neutral | 0.73 | 447 | 1.00 | KDEF |
| M19 | M | Neutral | 0.71 | 447 | 1.00 | KDEF |
| M20 | M | Neutral | 0.06 | 447 | 0.87 | KDEF |
| M21 | M | Neutral | 0.16 | 447 | 0.96 | KDEF |
| M22 | M | Neutral | 0.32 | 447 | 0.78 | KDEF |
| M23 | M | Neutral | 0.25 | 447 | 0.81 | KDEF |
| M24 | M | Neutral | 0.50 | 447 | 0.99 | KDEF |
| M25 | M | Neutral | 0.71 | 447 | 0.99 | KDEF |
| M26 | M | Neutral | 0.43 | 447 | 0.92 | KDEF |
| M27 | M | Neutral | 0.18 | 447 | 0.94 | KDEF |
| M28 | M | Neutral | 0.60 | 447 | 0.90 | KDEF |
| M29 | M | Neutral | 0.26 | 447 | 0.99 | KDEF |
| M30 | M | Neutral | 0.41 | 447 | 0.81 | KDEF |
| M31 | M | Neutral | 0.86 | 447 | 0.98 | KDEF |
| M32 | M | Neutral | 0.05 | 447 | 0.96 | KDEF |
| M33 | M | Neutral | 0.22 | 447 | 0.91 | KDEF |
| M34 | M | Neutral | 0.60 | 447 | 0.94 | KDEF |
| M35 | M | Neutral | 0.77 | 447 | 1.00 | KDEF |
| Mean |  |  | 0.59 |  | 0.90 |  |

*Note.* Image ID = picture ID from a particular dataset; F = female, M = male; N = number of human raters who judged the neutral face; FR = FaceReader; WSEFEP = *Warsaw Set of Emotional Facial Expression Pictures* (Olszanowski, et al., 2015), KDEF = *Karolinska Directed Emotional Faces* (Lundqvist et al., 1998).

**Appendix B**

Here, further information is provided on how accuracy scores for humans were arrived at in each of the datasets. The excerpts from specific rating procedure for each of the datasets is provided below.

**KDEF**

In order to simplify the rating procedure and to avoid overloading the participants, the 490 pictures were randomly divided into 12 slide shows. Eleven slide shows contained 41 pictures, and the remaining one had 39 pictures. Each of the KDEF pictures was rated by 64 participants. After deleting missing values and invalid answers (0.2%), each of the 490 KDEF pictures was rated by a minimum of 60 and a maximum of 64 participants (M=63.89; SD=0.39) on emotion, intensity, and arousal. (p. 1098, Goeleven et al., 2008)

**WSEFEP**

(…) Independent judges classified photographs into emotional categories and evaluated the intensity and purity of expression. Judges were asked to classify and evaluate individual photographs by pointing their mouse at a particular field in “the circle of emotions,” adapted based on Plutchik's circumplex model of emotions (1980). (…) The users could point at: (1) center of the field of a particular emotion category (which equally meant that in their opinion expression shown by the person in the photography is “clear”) or (2) field borders (which meant that the emotion is a mix of two neighboring emotions). Emotion intensity was measured at the same time: (1) showing the area closer to the circle meant low intensity of the expressed emotion or (2) closer to borders of the circle - high intensity of the expressed emotion. Emotional fields were all colored gradient gray, with darker areas at the borders between different emotions and brighter in the middle of the field. The area in the center of the circle meant neutral face expression. Additionally, the button named “other emotion” was located on the right site of the screen in case the participant would not be able to recognize the emotion. Fields outside of the wheel borders were deactivated, so only answers within the wheel or presses of the “other emotion” button were collected and allowed participants to continue to another trial. (…) After receiving an instruction with examples, each participant evaluated 20 photographs randomly chosen from the database (p. 4, Olszanowski et al. 2015).
